# Management of a Comminuted Patellar Fracture in a Child Using a Titanium Claw Plate: A Case Report

**DOI:** 10.7759/cureus.87083

**Published:** 2025-06-30

**Authors:** Chaimae Ben Driss, Mounir Erraji, Hamza Bensaghir, Houda Oubejja, Fouad Ettayebi

**Affiliations:** 1 Department of Pediatric Surgical Emergency, Children's Hospital of Rabat, Mohammed V University, Rabat, MAR

**Keywords:** case report, comminuted patellar fracture, knee surgery, pediatric trauma, titanium claw plate

## Abstract

Comminuted patellar fractures are rare in pediatric populations, often resulting from high-energy trauma such as sports or road accidents. Surgical management is essential to restore the articular surface and extensor mechanism of the knee. However, achieving anatomical reduction and rigid fixation can be challenging, potentially leading to poor functional outcomes.

We report the case of a 12-year-old boy who sustained a comminuted patellar fracture following a motorcycle accident. The patient presented with significant knee swelling, pain, and inability to bear weight. Radiographs and a CT scan confirmed the diagnosis. Surgical fixation was performed using a titanium claw plate, a novel approach in pediatric cases. Postoperative rehabilitation led to full knee mobility within three months.

Traditional methods such as tension band wiring are associated with complications such as skin irritation, loss of fixation, and symptomatic hardware. The titanium claw plate offers advantages such as shape memory effect, superelasticity, and biocompatibility, making it a promising alternative for pediatric patellar fractures.

Comminuted patellar fractures in children require surgical intervention to ensure optimal functional outcomes. The use of a titanium claw plate represents an innovative and effective treatment option, as demonstrated in this case. Further studies are needed to validate its long-term efficacy.

## Introduction

Comminuted patellar fractures are exceptionally rare in pediatric populations, accounting for less than 1% of all pediatric fractures [1]. These injuries typically result from direct trauma, such as sports-related accidents or road traffic collisions [2]. Unlike adults, children have a thicker periosteum and more resilient cartilage, which often leads to lower complication rates [3]. However, displaced comminuted fractures involving the articular surface require surgical intervention to restore knee function and prevent long-term disability [4].

The primary challenge in managing these fractures lies in achieving anatomic reduction and securing stable fixation, as the patellar fragments are often small and fragile. Traditional techniques, such as modified tension band wiring (TBW), involve passing Kirschner wires through the patella and securing them with a figure-of-eight stainless steel wire. While effective, this method often requires hardware removal due to irritation. However, these methods are associated with complications such as skin irritation, loss of fixation, and the need for hardware removal [5].

This case report presents the successful management of a comminuted patellar fracture in a 12-year-old boy using a titanium claw plate, a novel approach in pediatric trauma surgery. We discuss the advantages of this technique and its potential as an alternative to traditional methods.

## Case presentation

A 12-year-old boy with no significant medical history was admitted to the pediatric surgical emergency unit following a motorcycle accident. The patient sustained a direct trauma to the knee while in a flexed position, resulting in immediate pain, swelling, and functional disability.

Clinical examination

On admission, the patient exhibited significant swelling and tenderness in the knee region. The patellar tap test was positive, indicating hemarthrosis. No open wounds or neurovascular deficits were observed.

Imaging studies

**Figure 1:**
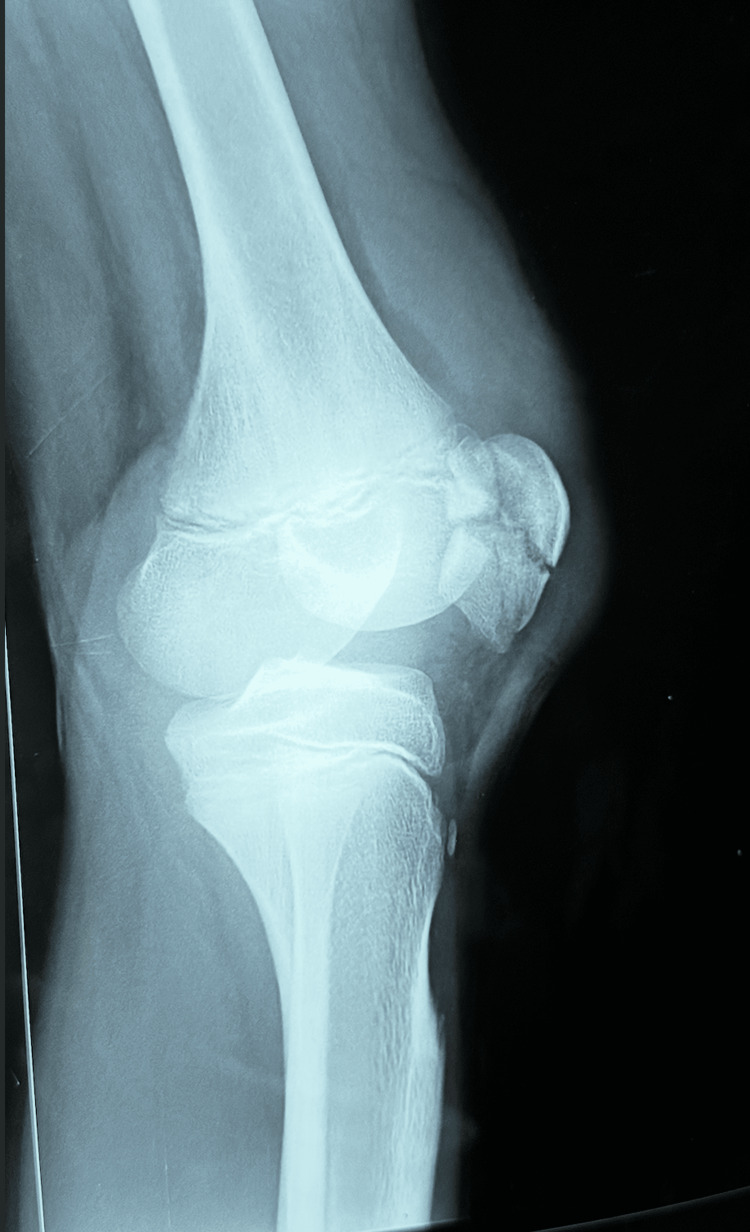
Preoperative X-ray showing a comminuted patellar fracture

Radiographs revealed a displaced comminuted patellar fracture (Figure 1).

**Figure 2:**
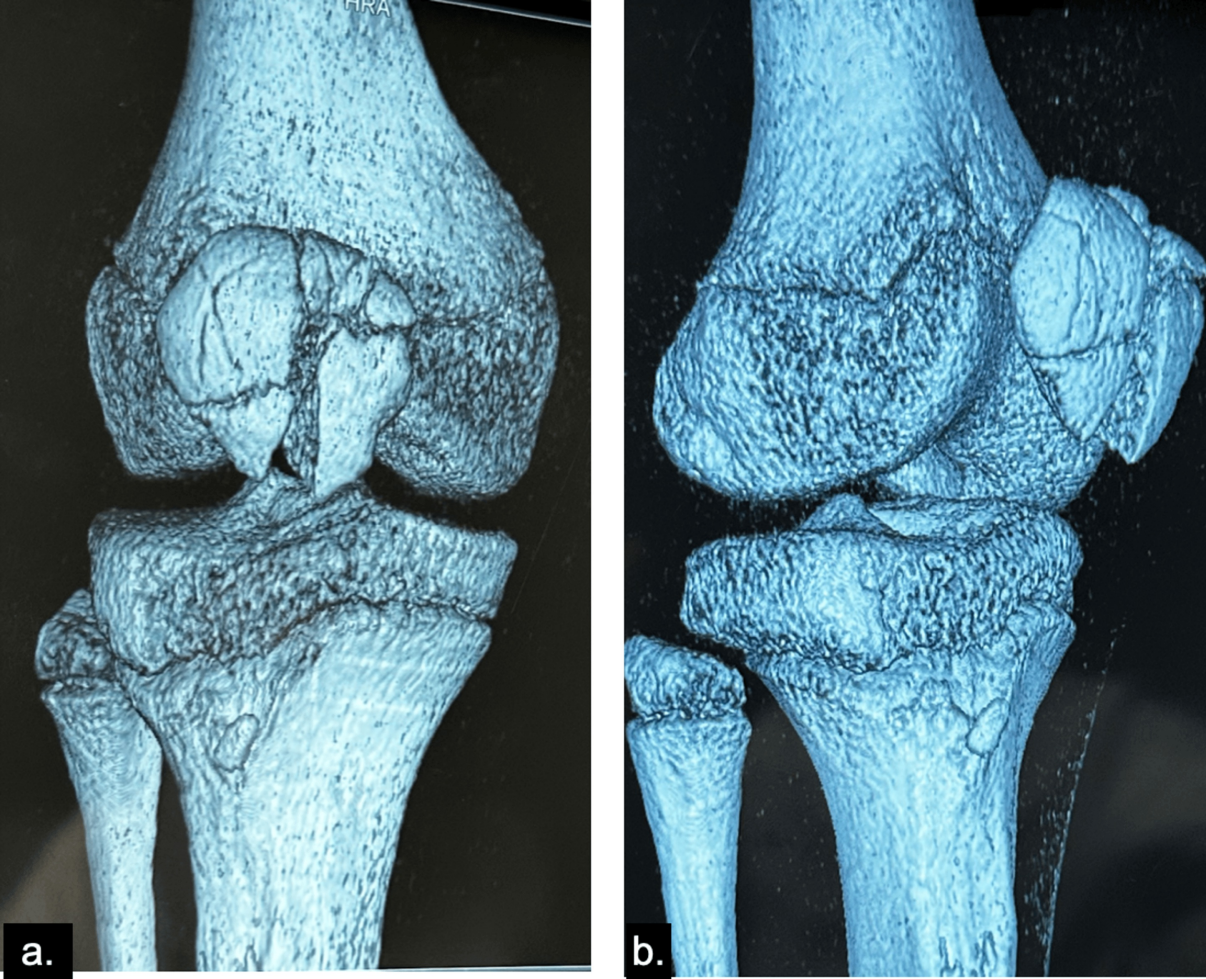
CT scan confirming the comminuted fracture pattern a. Frontal and b. Lateral

A CT scan was performed to further characterize the fracture pattern and confirm the diagnosis (Figure 2).

Surgical management

**Figure 3:**
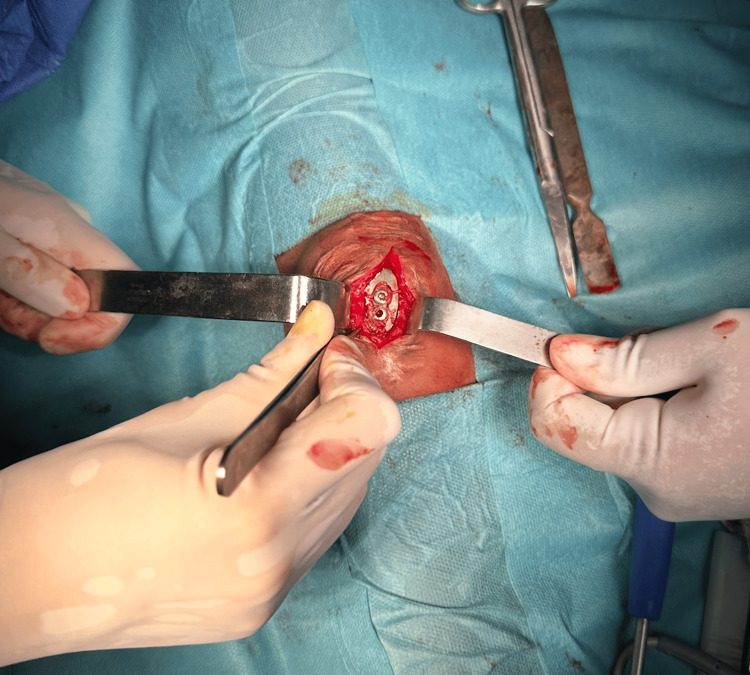
Intraoperative image of titanium claw plate fixation

The patient underwent surgical fixation using a titanium claw plate (Figure 3). This technique was chosen due to the fracture’s comminution and the need for stable, low-profile fixation.

Under general anesthesia, the fracture fragments were reduced and stabilized using the claw plate, which was secured through the quadriceps and patellar tendons. Intraoperative X-rays confirmed anatomical reduction and proper placement of the implant.

Postoperative course

**Figure 4:**
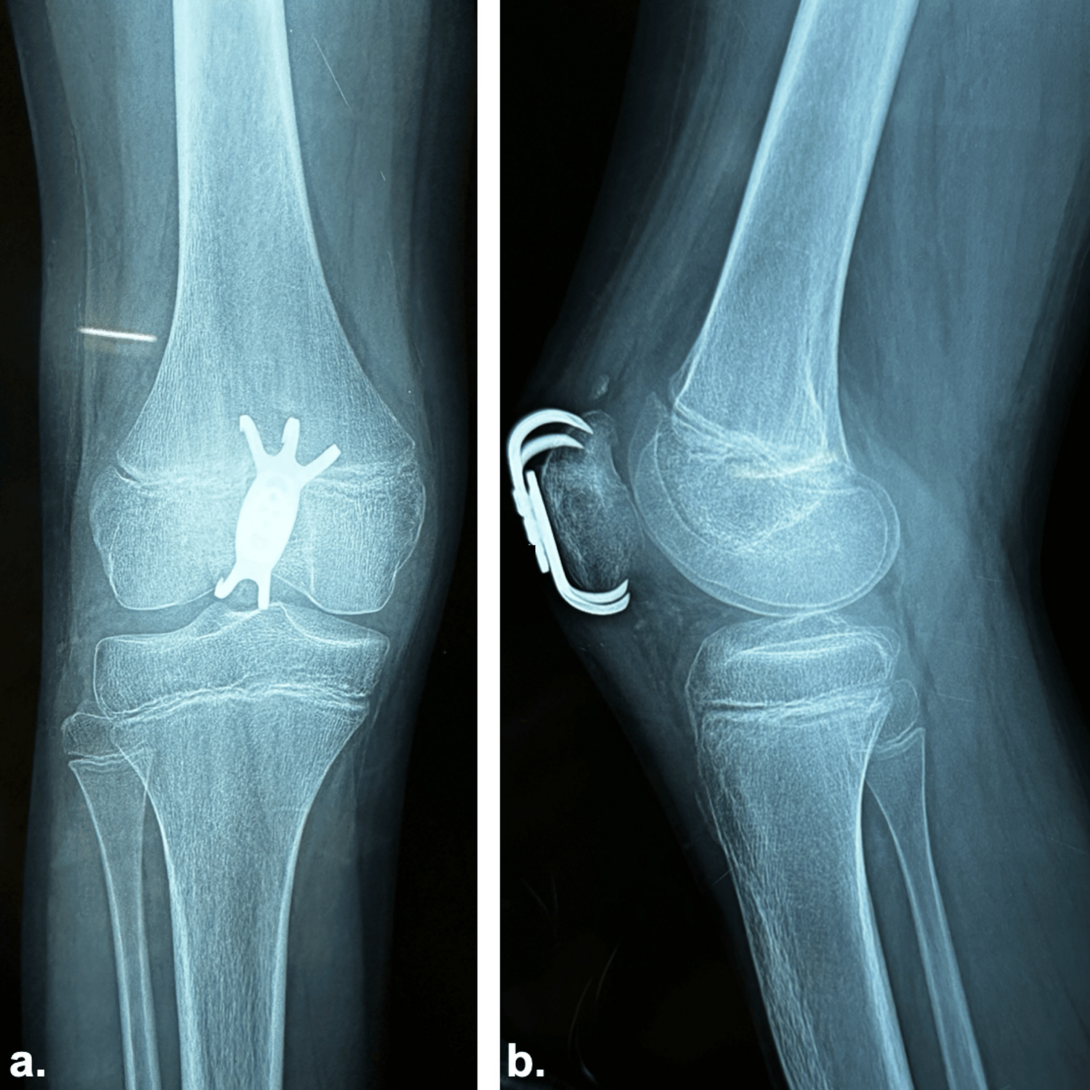
Postoperative X-ray at three months demonstrating fracture healing and restored patellar alignment a. Frontal view and b. Lateral view

The knee was immobilized in a splint for two weeks to allow wound healing. Passive range-of-motion exercises were initiated after splint removal, followed by active exercises at three weeks. Full weight-bearing was permitted one month after the procedure. At three months postoperatively, the patient achieved full knee mobility without pain or functional limitations (Figure 4).

## Discussion

Comminuted patellar fractures in children are rare but present significant challenges due to the small size and fragility of the patellar fragments. These fractures typically result from high-energy trauma, such as road traffic accidents or sports injuries, and often involve the articular surface, requiring surgical intervention to restore knee function and prevent long-term complications [1,4].

Challenges in pediatric patellar fractures

The management of comminuted patellar fractures in children differs from that in adults due to the unique anatomical and physiological characteristics of pediatric bone. Children have thicker periosteum and more resilient cartilage, which can aid in healing, yet may complicate surgical fixation due to the small size of the fragments [5]. Traditional techniques, such as modified TBW supplemented with Kirschner wires or cannulated lag screws, have been the mainstay of treatment. Unlike adult bone, pediatric bone has greater elasticity and remodeling potential, necessitating implants that minimize growth disturbance. However, these methods are associated with several limitations, including intraoperative difficulty in wire manipulation, postoperative complications like skin irritation, loss of fixation, and the need for hardware removal [6,7].

The titanium claw plate: a novel approach

The titanium claw plate used in this case represents a promising advancement in the management of comminuted patellar fractures. This implant belongs to the category of "smart materials," which are characterized by shape memory effect, superelasticity, excellent fatigue behavior, corrosion resistance, and high damping capacity [7]. These properties make the claw plate particularly suitable for pediatric cases, where secure fixation of small fragments is critical [8].

The claw plate’s design allows it to penetrate the quadriceps and patellar tendons, providing stable fixation without compromising the soft tissue. Intraoperative X-rays confirmed anatomical reduction and proper placement of the implant, which is essential for restoring the patellofemoral joint’s normal alignment [9]. Postoperatively, the patient achieved full knee mobility within three months, demonstrating the effectiveness of this technique.

Advantages over traditional methods

Compared to TBW, the titanium claw plate offers several advantages. First, it eliminates the need for complex wire manipulation, reducing intraoperative time and technical difficulty [10]. Second, its biocompatibility minimizes the risk of adverse reactions, such as infection or allergic responses, which are common with metallic implants [11]. Third, the claw plate’s design reduces the likelihood of symptomatic hardware, since its low-profile design and tendon-integrated fixation decrease soft tissue irritation, often necessitating removal in TBW cases [12]. The claw plate’s low-profile design and tendon-integrated fixation decrease soft tissue irritation.

Limitations and future directions

While the titanium claw plate shows promise, its use in pediatric patellar fractures is still relatively new, and long-term data are limited. Potential limitations include the cost of the implant and the need for specialized surgical expertise [13]. Future studies should focus on larger cohorts and longer follow-up periods to evaluate the long-term outcomes of this technique. Additionally, comparative studies between the claw plate and traditional methods would provide valuable insights into its relative efficacy and safety [14].

Broader implications for pediatric trauma surgery

The successful application of the titanium claw plate in this case highlights the role of innovative approaches in advancing pediatric trauma care. As children have unique anatomical and physiological needs, tailored approaches are essential to optimize outcomes [15]. The use of advanced materials and techniques, such as the claw plate, may represent a step forward in addressing the challenges of pediatric fractures.

Furthermore, this case underscores the need for multidisciplinary collaboration in managing complex pediatric injuries. Orthopedic surgeons, radiologists, and rehabilitation specialists must work together to ensure comprehensive care, from diagnosis to postoperative recovery [16].

## Conclusions

Comminuted patellar fractures in children are rare but require prompt and effective surgical intervention. The titanium claw plate offers a promising alternative to traditional methods, with advantages such as secure fixation, biocompatibility, and reduced need for hardware removal. This case illustrates the successful use of this technique in a pediatric patient, with favorable functional outcomes observed at the three-month follow-up. However, further research including prospective and comparative studies is needed to validate its long-term efficacy and safety in larger populations.

## References

[1] Springorum HP, Siewe J, Dargel J, Schiffer G, Michael JW, Eysel P (2011). Classification and treatment of patella fractures (Article in German). Orthopade.

[2] Melvin JS, Mehta S (2011). Patellar fractures in adults. J Am Acad Orthop Surg.

[3] Gwinner C, Märdian S, Schwabe P, Schaser KD, Krapohl BD, Jung TM (2016). Current concepts review: fractures of the patella. GMS Interdiscip Plast Reconstr Surg DGPW.

[4] Banks KE, Ambrose CG, Wheeless JS, Tissue CM, Sen M (2013). An alternative patellar fracture fixation: a biomechanical study. J Orthop Trauma.

[5] Carpenter JE, Kasman RA, Patel N, Lee ML, Goldstein SA (1997). Biomechanical evaluation of current patella fracture fixation techniques. J Orthop Trauma.

[6] Smith ST, Cramer KE, Karges DE, Watson JT, Moed BR (1997). Early complications in the operative treatment of patella fractures. J Orthop Trauma.

[7] Gosal HS, Singh P, Field RE (2001). Clinical experience of patellar fracture fixation using metal wire or non-absorbable polyester—a study of 37 cases. Injury.

[8] Ansari S, Barman S, Raja BS (2024). Pediatric patella fractures - a systematic review. J Orthop.

[9] Wang X, Xu S, Zhou S (2016). Topological design and additive manufacturing of porous metals for bone scaffolds and orthopaedic implants: a review. Biomaterials.

[10] Lazaro LE, Cross MB, Lorich DG (2014). Vascular anatomy of the patella: implications for total knee arthroplasty surgical approaches. Knee.

[11] Böstman OM, Pihlajamäki HK (2000). Adverse tissue reactions to bioabsorbable fixation devices. Clin Orthop Relat Res.

[12] Matejcić A, Smiljanić B, Bekavac-Beslin M (2008). The use of memory shape staples in orthopedic surgery (Article in Croatian). Acta Clin Croat.

[13] Bickel S, Jensen KO, Klingebiel FK (2024). Clinical and functional outcomes of locked plating vs. cerclage compression wiring for AO type C patellar fractures- a retrospective single-center cohort study. Eur J Trauma Emerg Surg.

[14] Gardner MJ, Griffith MH, Lawrence BD, Lorich DG (2005). Complete exposure of the articular surface for fixation of patellar fractures. J Orthop Trauma.

[15] Skaggs DL (2014). Rockwood and Wilkins’ Fractures in Children. 8th ed.

[16] Ibrahim S (2015). Tachdjian's Pediatric Orthopaedics: from the Texas Scottish Rite Hospital for Children. Malays Orthop J.

